# Effects of Mountain-Basin System on Chemical Composition, Antioxidant Activity and Volatile Flavor Substances of Cabernet Sauvignon Wines in Xinjiang Region, China

**DOI:** 10.3390/foods14071086

**Published:** 2025-03-21

**Authors:** Junbo Zhang, Yapeng Qi, Ruiyang Han, Miao Cui, Feifei Gao, Ping Wang, Qinming Sun

**Affiliations:** 1 Key Laboratory of Agricultural Product Processing and Quality Control of Specialty (Co-Construction by Ministry and Province), School of Food Science and Technology, Shihezi University, Shihezi 832000, China; zhangjunbo1205@163.com (J.Z.); qiyapeng6013@163.com (Y.Q.); 20221011023@stu.shzu.edu.cn (R.H.); 17861506386@163.com (M.C.); gaofeifei@shzu.edu.cn (F.G.); 2 Key Laboratory for Food Nutrition and Safety Control of Xinjiang Production and Construction Corps, School of Food Science and Technology, Shihezi University, Shihezi 832000, China; 3 Agricultural College, Shihezi University, Shihezi 832003, China

**Keywords:** mountain-basin system, climate, terrain, cabernet sauvignon wine, antioxidant activity, volatile compound

## Abstract

To investigate the effect of mountain-basin system on wine quality, four different regions were selected according to altitude and latitude. This work analyzed the differences in physicochemical properties, organic acids, monomeric phenols, antioxidant activity and volatile compounds of Cabernet Sauvignon wine between four regions. Comparative analysis revealed that there were significant differences in alcohol content and pH, respectively. Malic acid in organic acids was the main acid to distinguish the four regions. Correlation analysis showed that there was a significant correlation between physicochemical properties and climatic conditions in the four regions. There were significant differences in most of the monomeric phenols, and the antioxidant capacity was also significantly different. A total of 60 volatile compounds were detected, including 11 key volatile compounds, and there were significant differences in the composition of wines in the four regions. Methyl salicylate, ethyl caprate and ethyl hexanoate were the characteristic aromas in mountain front (MF) and intermontane basin (IB) regions, decanal was the characteristic aroma in sloping field (SF) region, and ethyl butyrate was the characteristic aroma in near desert (ND) region. This study further clarified the influence of climate and terrain on wine quality, and provided a better theoretical basis for the fine management of small producing areas.

## 1. Introduction

The mountain-basin system (MBS) is a unique and complex geomorphological structure composed of the vertical belt system of mountain vegetation and the vegetation zone of desert basin. The system includes mountain, piedmont inclined plain and alluvial plain [1]. Due to the differences in altitude and topography, there are significant differences in natural environment and climatic conditions in different regions of the mountain-basin system. Thus, wine grape cultivation areas with different terroir characteristics were formed. The main factors affecting regional differences are geographical characteristics, including climate, temperature, humidity, lighting, geology, terrain, altitude, and soil [2].

Wine is a popular alcoholic beverage. The quality of wine mainly depends on the quality of grape berries. Studies have shown that the quality of grape berries is affected by factors such as soil, climate, and variety. Different climatic conditions formed under different terrain conditions are related to the formation of grape fruit quality [3]. Moreover, the quality and typicality of wine will also be affected by temperature, humidity, light, altitude and other factors [4]. These abiotic factors directly or indirectly regulate the biosynthesis and degradation of primary (sugars, organic acids, etc.) and secondary (phenols and volatile flavor compounds and their precursors) metabolites by regulating their biosynthetic pathways, or by affecting the physiology and phenology of grapes [5]. The difference of accumulated temperature in different regions will lead to the difference of grape quality. High temperature will induce enzyme reaction, increase sugar content and decrease acidity [6]. In addition, moisture plays a key role in grape growth. The growth environment of different humidity can provide different water content for grapes [7]. Studies have shown that the decrease of water will make the volume of grape smaller, the concentration of sugar will increase, and the proportion of peel will increase, and the content of phenols in grape will increase [5]. Therefore, due to the influence of meteorological factors, the mountain basin system may affect the quality of wine.

The quality of wine varies greatly in vineyards in different regions. The main indexes to evaluate the quality of wine are sugar, acid, color, tannin, polyphenol, antioxidant and aroma. Phenolic compounds contribute greatly to the color, taste and palatability of wines. Furthermore, these compounds are recognized for their capacity to neutralize surplus free radicals and regulate reactive oxygen species (ROS) homeostasis within biological systems [8]. Nicholas et al. found that temperature also has an important effect on the synthesis and accumulation of phenolic substances [9]. Balint et al. found that the decrease of environmental humidity during grape version would reduce the water absorption of berries [7]. The content of anthocyanins and total phenols in grape berries was significantly increased, and the color and taste of wine were greatly improved. Jiang et al. conducted quantitative analyses of phenolic compounds and antioxidative potential in wines from four major viticultural regions across China [10]. It was found that the amount of phenolics and antioxidant capacity in wines from these regions varied greatly, depending on the environmental factors of grape growth. Growing environment (climate, light and altitude) is one of the many factors that affect wine aroma. He et al. studied the relationship between wine aroma components and topography in four plots on the same slope, and discussed the deep relationship between them [11]. They found that wines from four different vineyard plots had significant differences in the olfactory characteristics of floral and fruity aromas, which were mainly related to esters with high aroma activity.

Cabernet Sauvignon (*Vitis vinifera* L.), originated from France is widely planted all over the world because of its strong adaptability to the environment [12]. It is the world’s largest wine grape variety. Cabernet Sauvignon is also the largest cultivated variety in Xinjiang, China’s main producing area. Nowadays, many people have studied the differences in wine producing areas between large regions. However, few studies have focused on the small regions under the influence of the mountain-basin system, as well as the fine division of small regions within the production region and the differences in wine flavor substances. Based on the altitude and latitude, this study selected four regions along the Manas River Basin in the northern foot of the Tianshan Mountains. Then the effects of four regional climates on the flavor and quality of wine were explored. Therefore, we measured the meteorological indicators of the four regions, and measured the physicochemical properties, organic acids, polyphenols, antioxidant properties and volatile compounds of the wine. This study starts with factors such as climate and topography to find differences in wine flavor and quality. In order to provide a theoretical basis for the fine division of typical wine producing areas in Xinjiang.

## 2. Materials and Methods

### 2.1. Sampling Sites and Method

In this study, four sampling points were selected along the Manas River Basin in Xinjiang, China according to altitude and latitude, namely mountain front (MF, 86°6′25″ E, 44°2′15″ N, 617 m), sloping field (SF, 85°54′38″ E, 44°16′15″ N, 492 m), intermontane basin (IB, 86°2′15″ E, 44°30′25″ N, 384 m), and near desert (ND, 85°18′13″ E, 44°42′12″ N, 296 m). The interval of each sampling site is about 10 km, and the altitude difference is about 100 m. The vineyards of each sampling sites are uniformly planted and managed by Chateau Changyu Baron Balboa, north-south direction.

In each region, a relatively flat Cabernet Sauvignon vineyard was selected. The five-point sampling method was selected, and an area was selected as the sampling point at the center point of the central area of the vineyard and the center point of the four corners. In the grape harvest period, a cluster of grapes was collected in the upper, middle and lower parts of the grape plants in the selected area. The grapes were mixed and transported back to the laboratory and divided into two parts. One part was divided into three groups, and the other part was stored at −20 °C.

### 2.2. Wine Fermentation

Healthy grape berries without obvious plant diseases were selected, crushed, and placed in a 5-L sterile glass fermenter. The soluble solids and pH of grape juice were detected as the characterization of grape maturity. Broken grape juice was added with 20 mg/L pectinase (Zhejiang Yinuo Biotechnology Co., Ltd., Shaoxing, China) and 30 mg/L sulfur dioxide (potassium metabisulfite) and macerated for 24 h at 4 °C in the dark. The fermenters were placed at room temperature, and the grape juice underwent alcoholic fermentation after the addition of Excellence XR yeast (Bordeaux, France). Throughout the process, daily monitoring of soluble solids, temperature, specific gravity, and pH of grape juice were monitored. When the measured value remains stable, the fermentation is considered to be completed. Finally, the skin residue was separated and 30 mg/L sulfur dioxide was added to the wine for better storage. The wines were stored in 750 mL bottles for further analysis.

### 2.3. Determination of Meteorological Indexes and Physiochemical Properties

The temperature and humidity of four vineyards were detected by Remote Environmental Detector (S21A, Xuzhou Farah Electronic Technology Co., Ltd., Xuzhou, China) every 1 h, and the temperature and humidity within 50 days before harvest were detected. A probe head is placed on the north and south respectively, and a probe head is placed on the surface. Three sets of data are repeated.

The soluble solids content (SSC), alcohol content (AC), pH value, total sugar (TSC) and titratable acid (TA) of wine were determined. Soluble solids content was measured by hand-held refractometer (WS108, Hebei Runlian Technology Development Co., Ltd., Baoding, China). Alcohol content is determined by the alcohol meter (HMM-I, Shanghai Precision Instrument Co., Ltd., Shanghai, China) method. The pH value was measured using a pH meter (pHS-3C, Shanghai Yitian Scientific Instrument Co., Ltd., Shanghai, China). Reducing sugar was determined by 3,5-dinitrosalicylic acid method. The titratable acid content was determined by direct titration method [13].

### 2.4. Determination of Organic Acids

The determination of organic acid components was performed by high performance liquid chromatography (Agilent, Waters, Milford, MA, USA), which was modified according to the previously reported method [14]. The wine samples 1 mL were centrifuged (12,000 r/min) and then proportionally diluted and filtered through a 0.45 µm membrane. The chromatographic conditions were as follows: PDA detector, C18 column (5 µm, 4.6 mm × 250 mm; diamonsil Plus Technology, Beijing, China). The mobile phase A and B was methanol and 0.1 % phosphoric acid water, respectively. The column temperature was 30 °C, the injection volume was 10 µm, and the detection wavelength was 210–400 nm. The sample elution method was isocratic elution, A phase was 0.04 mL/min, B phase was 0.96 mL/min. The content of organic acids in the sample was calculated by substituting the detected peak area into the standard curve.

### 2.5. Determination of Phenolic Compounds

Total phenolic content (TPC) was quantitatively evaluated through Folin-Ciocalteu method [15]. The absorbance was measured at 765 nm, and the results were expressed as gallic acid equivalent value (mg/L). The content of tannin (TC) was determined by Folin-Dennis method [16]. The absorbance was measured at a wavelength of 650 nm, and the corresponding tannin content was calculated from the standard curve by the absorbance. The content of anthocyanin (TAC) was determined by pH differential method [17], and the results of the two diluted samples were measured at 510 nm and 700 nm, respectively. The total flavonoid (TFC) was determined at a wavelength of 510 nm according to the method of Mitic et al. [18], and the results were expressed as the equal value of rutin. Each treatment was repeated three times.

The monomer phenolic compounds in wine were extracted by the previous methods with slight modifications [19]. 5 mL wine sample and equal volume of ethyl acetate were added and mixed with a spiral oscillator for 30 s, extracted ultra sonically for 30 min, and centrifuged for 15 min at 10,000 rpm, 4 °C. The extraction processes were repeated for 3 times. The combined supernatant was evaporated by rotary evaporator (RE–2000A, Shanghai Yarong Biochemical Instrument Co., Ltd., Shanghai, China) at 40 °C. The residue was dissolved in 5 mL methanol and filtered with 0.22 µm membrane for storage.

The monomer phenolic compounds were determined by high performance liquid chromatography (Agilent, Waters, Milford, MA, USA). The detection conditions are as follows: the detector is PDA detector, and the chromatographic column was C18 column. The mobile phase A and B were methanol and acetic acid water (1 %), respectively. The column temperature was 30 °C, the injection volume was 10 µm, and the detection wavelength was 210–400 nm. The gradient elution procedure was as follows: 5 % A at 0 min, 30 % A at 20 min, 50 % A at 40 min, 95 % A at 55 min, 5 % A at 60 min, and the total flow rate with 1 mL/min. The peak time and chromatogram of the sample and the standard were compared for qualitative analysis, and the phenolic compounds were quantitatively analyzed by the standard curve.

### 2.6. Determination of Antioxidant Activities in Vitro

DPPH assay was slightly modified according to the method proposed by Jiang et al. [10]. 0.1 mL diluted 10 times sample was mixed with add 3.9 mL DPPH working solution, and place in darkness for 30 min. Then, the absorbance of the mixed solution was measured at 517 nm. The results were expressed as mg Trolox/L equivalent value.

ABTS was determined using the previous method [20]. The 7 mmol/L ABTS solution was mixed with an equal volume of 2.6 mmol/L potassium persulfate solution and diluted. The 0.1 mL diluted wine sample was added to 3.9 mL ABTS solution and reacted for 10 min under dark conditions. The absorbance was measured at a wavelength of 734 nm. The results were expressed as mg Trolox/L equivalent value.

CUPRAC reduction capability was detected by the method [21]. 0.1 mL of 10-fold diluted wine sample was taken, and 1 mL of 5 mmol/L copper sulfate solution, 1 mL of 3.75 mmol/L neocuproine, 1 mL of 1 mol/L ammonium acetate buffer and 1 mL distilled water were added in turn. The total volume is 4.1 mL. The absorbance was measured at 450 nm after mixing and reacting for 30 min. The data were quantified and expressed in Trolox equivalent units.

FRAP reduction capability was detected via the method [22]. The 0.1 mL wine sample was added to 3 mL of FRAP working solution, and then 0.3 mL of ultrapure water was added. The mixed samples were reacted in water bath (37 °C, 10 min), and the absorbance was measured at 593 nm. The data were quantified and expressed in Trolox equivalent units.

### 2.7. Determination of Volatile Compounds

Volatile compounds in wines were extracted referring to the previous method [23]. The volatile compounds were extracted by headspace solid phase microextraction technique (HS-SPME). 5 mL wine sample and 1 g saturated sodium chloride were added to the headspace bottle, 2 µL 3-octanol was added as internal standard. The sample was equilibrated for 15 min (45 °C, 800 r/min) and extracted for 40 min (45 °C, 800 r/min). Then the sample was injected and desorbed in a split-less mode at 230 °C for 5 min.

The aroma substances in wine were characterized by GC-MS detector (Agilent 7000D, Technologies Inc., Santa Clara, CA, USA) and separated by polar capillary column (HP-Innowax, 30 m × 0.25 mm × 0.25 µm, Agilent Technologies Inc., Santa Clara, CA, USA). The initial temperature of the column oven was set to 50 °C, and the temperature was increased at a constant flow rate of 1 mL/min and maintained for 5 min. The temperature was initiated with a programmed heating gradient of 3 °C/min until reaching 85 °C, followed by sequential elevation phases at 4 °C/min to 180 °C and accelerated ramping (10 °C/min) culminating at 230 °C, and maintained for 5 min. The mass spectrometry scan rate was 5 times per second, and the range was 35–350 m/z.

The volatile compounds in wine were preliminarily identified by NIST 98 and Wiley 6 mass spectrometry libraries. The relative concentration of volatile compounds in wine was calculated based on the peak area of internal standard. Geographical typicity of wine volatiles was quantitatively assessed through odor activity value (OAV), and the key contributing substances (OAVs ≥ 1) were identified. The OAV is calculated from the ratio of the concentration of volatile substances in the aqueous solution to its corresponding sensory detection threshold [24].

### 2.8. Statistical Analysis

All three biological replicates were collated using Microsoft Excel 2019, and the statistical comparison of variance between groups was performed using the SPSS 20.0 (IBM Chicago, IL, USA) to analyze the significant differences in the data of each group at the 5 % level (*p* < 0.05). Origin 2022 was used to generate histograms and visual graphs of correlation coefficients. TBtools v2.038 was used to generate cluster heat maps. The distribution of wines from different producing areas were analyzed by principal component analysis with MetaboAnalyst (https://www.metaboanalyst.ca/) (accessed on December 2024). Cytoscape v3.10.2 was used to draw the correlation network diagram between climatic factors and wine quality.

## 3. Results and Discussion

### 3.1. Differences in Meteorological Indicators in Different Regions

In this study, the differences in altitude between 296 m to 617 m and latitude with an interval of 10 km influenced the meteorological indicators such as average temperature, humidity and diurnal amplitude in the four regions. In order to determine the effect of meteorological parameters on wine quality, we measured the temperature and humidity in different regions (Table 1). The results showed that there were significant differences in meteorological environment in these regions. During the period from color change to ripening of the grape berries, the average temperature of the four regions ranged from 17.27 °C to 18.81 °C, the diurnal temperature difference ranged from 18.86 °C to 19.88 °C, and the relative humidity ranged from 66.69 % to 68.99 %. The change trend of temperature gradually increases with the decrease of altitude, and the change trend of water humidity decreases with the decrease of altitude. The diurnal temperature difference in the IB region was the highest, and the diurnal temperature difference in the SF region was the lowest.

### 3.2. Differences in Physicochemical Properties of Wine from Different Regions

**Figure 1:**
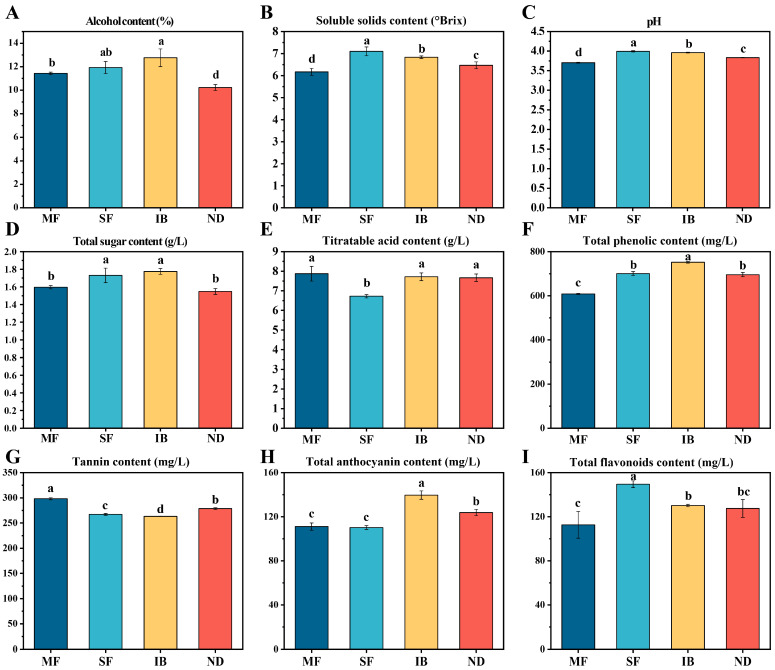
The differences in physicochemical properties of wines from four regions. (**A**) Alcohol content. (**B**) Soluble solids content. (**C**) pH. (**D**) Total sugar content. (**E**) Titratable acid content. (**F**) Total phenolic content. (**G**) Tannin content. (**H**) Total anthocyanin content. (**I**) Total flavonoids content. The letters indicate significant between different regions (*p* < 0.05).

To compare the differences in physicochemical properties of wine from different regions, this study determined alcohol content, soluble solids, pH, residual sugar and titratable acid of wine. According to Figure 1, there were significant differences in alcohol content, soluble solids and pH in different regions (*p* < 0.05). The content of titratable acid in MF region was the highest, and the change trend of pH affected by acidity was opposite to that of titratable acid. The alcohol content and residual sugar content in IB region were the highest, and the residual sugar content was also the highest. Alcohol was transformed from reducing sugar in grape berries, so the alcohol content and residual sugar were affected by the initial sugar content of grape berries [6]. As shown in Table S1, the difference between SSC and pH in the four regions early fermentation was almost the same as that on the 7th day after fermentation. The SSC decreased during the fermentation process. The pH trend decreased first and then increased, and finally tended to be stable.

The main sources of phenolic compounds in wine are grape skins and seeds, which are affected by climate and terrain height [8]. There were significant differences in TPC, TC, TFC and TAC in wines from different regions (*p* < 0.05). The highest contents of TPC and TAC in IB were 752 mg/L and 140 mg/L, respectively (Figure 1F,H). It may be that the average temperature in the IB region is low and the diurnal amplitude is large. Because the higher the temperature at the maturity stage of wine grapes, the lower the anthocyanin content. The greater the diurnal amplitude, the higher the anthocyanin content [25]. In addition to the highest tannin content of 298 mg/L in MF, the other indexes were the lowest (Figure 1G). The TPC in the SF region was the highest, which was 149 mg/L (Figure 1I). This result is similar to the research results of Juliane et al. in the tropical planting area of Brazil. Their conclusion is that tannins and flavanols are higher at higher altitudes [26]. Different temperatures, moisture humidity and altitudes would affect the expression of some key genes to promote and inhibit activation mechanisms in anthocyanin and flavonoid metabolic pathways [5].

### 3.3. Correlation Analysis of Environmental Factors and Physicochemical Properties

**Figure 2:**
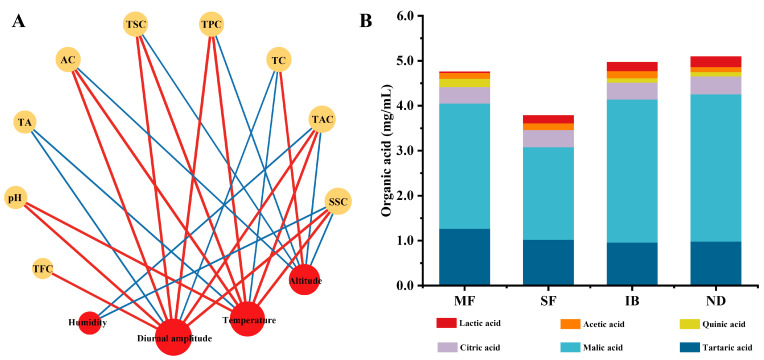
Network analysis reflected the co-occurrence relationship between wine physicochemical properties and meteorological parameters in different regions (**A**). Red circular nodes represent meteorological parameters, and yellow circular nodes represent physicochemical properties. The direct connection between nodes indicates a strong correlation. The color of the edge represents positive correlation (red) or negative correlation (blue). The stacking histogram reflected the difference of organic acids in wines from different regions (**B**).

Network analysis was conducted to explore the correlation between the basic physicochemical properties of wine and meteorological indicators (Figure 2A), based on the strong correlation coefficients (Pearson correlation coefficient, |ρ| ≥ 0.5; *p* < 0.05). Temperature was positively correlated with AC, SSC, pH, TSC, TPC, TAC, and negatively correlated with TA and TC. The increase of temperature may promote the increase of sugar content and the decrease of acid content in grapes [6]. Therefore, after brewing into wine, it may lead to an increase in alcohol and residual sugar, and a decrease in total acid content. Moisture humidity was negatively correlated with SSC and TAC. It is reported that the decrease of environmental humidity will reduce the water absorption of berries, significantly increase the anthocyanin content of grape berries, and greatly improve the color and taste of wine [7]. The diurnal temperature difference was positively correlated with AC, SSC, pH, TSC, TPC and TAC, and negatively correlated with TA and TC. Increased diurnal temperature difference will improve the quality of grape berries, and thus the quality of wine. Because within an optimal thermal range, photosynthetic activity intensifies with increased daylight temperatures, and the lower the nighttime temperature, the lower the respiratory consumption. Therefore, it provides the necessary resources and precursors for the synthesis of secondary metabolites in grape berries [5]. The Altitude was positively correlated with TC and negatively correlated with AC, SSC, TSC, TPC and TAC. Altitude generally affects the quality of grape berries by affecting the microclimate [27].

### 3.4. Organic Acids

The content and type of organic acids are important factors affecting the flavor, taste, color and stability of wine [28]. In this study, six organic acids including tartaric acid, malic acid, quinic acid, lactic acid, citric acid and acetic acid, were quantified in wines from different regions (Figure 2B). The content of tartaric acid and malic acid was the highest in the four regions. The content of tartaric acid was between 0.96 mg/mL and 1.27 mg/mL, accounting for 22.76 % of the total. The content of malic acid was between 2.065 mg/mL and 3.275 mg/mL, accounting for 60.79 % of the total. Malic acid is derived from grape berries, and its accumulation is affected by climate and environment. Among the four regions, the malic acid content in IB region was the highest, and that in SF region was the lowest, which may be due to the fact that the increase of temperature accelerated the accumulation of malic acid in the stage of grape berry development [29]. The content of tartaric acid was the highest in MF region and the lowest in IB region. The difference between tartaric acid in different regions is small, which may be due to the fact that tartaric acid is not easily affected by climatic conditions during grape ripening [28].

### 3.5. Analysis of Monomeric Phenol Content and Antioxidant Activity in Different Regions

**Figure 3:**
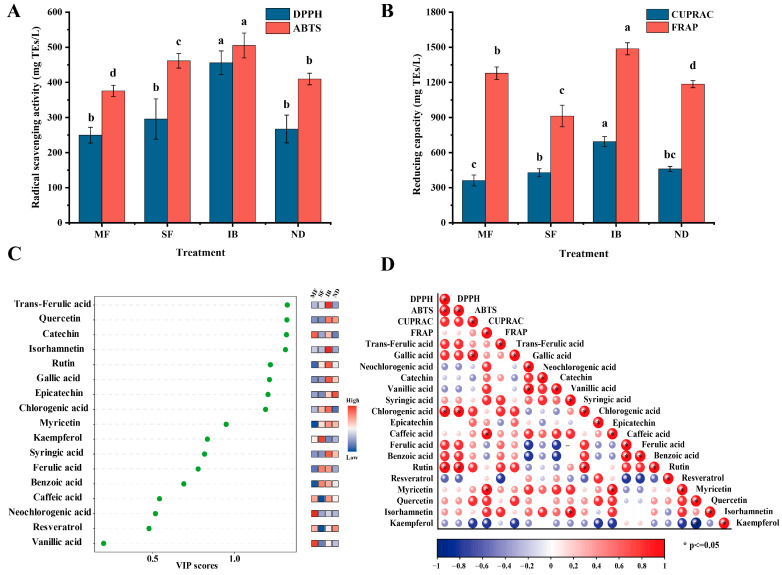
Differential analysis and correlation of antioxidant activity and monomer phenol content in wine samples from different regions. (**A**) DPPH and ABTS free radical scavenging ability. (**B**) CUPRAC and FRAP reduction ability. (**C**) VIP value plot and heat map. (**D**) The relationship between phenolic compounds and antioxidant activity. The letters indicate significant between different regions (*p* < 0.05).

In this study, 17 monomeric phenolic compounds were detected in wines from four regions, including 8 flavonoids and 9 non-flavonoids (Table S2). Among them, the contents of rutin and epicatechin were the highest, and there were significant differences in four regions (*p* < 0.05). According to the VIP value plot analysis (Figure 3C), the VIP values of trans ferulic acid, gallic acid, catechin, epicatechin, chlorogenic acid, rutin, quercetin, and isorhamnetin were all greater than 1. It shows that they are potential markers to distinguish the differences between different regions. The contents of gallic acid, rutin, quercetin and isorhamnetin were the highest in IB producing area, catechin was the highest in MF producing area, and epicatechin was the highest in ND producing area. Most of the high content of monomeric phenols in the four regions are flavonoids. Flavonoids account for a large proportion of phenolic compounds in red wine and can be considered as the most important quality-determining compounds in red wine [30].

The antioxidant activity of wine mainly comes from its own phenols, and the composition of phenols depend on grape varieties, the geographical characteristics of the vineyard, climate, production process and aging [10]. The DPPH and ABTS methods were used to study the radical scavenging ability of wines from different regions (Figure 3A). The ABTS free radical scavenging ability of the four regions was significantly different (*p* < 0.05). The IB region had the highest DPPH and ABTS radical scavenging abilities among the four regions, with 456 mg TEs/L and 505 mg TEs/L, respectively. This may be due to the higher phenolic content in the IB region than in other regions. Meanwhile, the reduction capacities of wines among different regions were investigated based on FRAP and CUPRAC methods, and there were significant differences in FRAP and CUPRAC reduction capacities among the four regions (Figure 3B). The FRAP reduction capacities of the four regions ranged from 913 mg TEs/L to 1487 mg TEs/L, and the CUPRAC reduction capacities ranged from 362 mg TEs/L to 694 mg TEs/L. The IB region had the highest CUPRAC and FRAP reduction capacities among the four regions, which were 694 mg TEs/L and 1487 mg TEs/L, respectively.

In order to study the correlation between monomeric phenols and antioxidant activity in wines from four regions, we analyzed based on the Pearson correlation coefficient matrix (Figure 3D). The results showed that these phenolic compounds had a strong correlation with the antioxidant capacity of wines from different regions. Among them, trans-ferulic acid, catechin, epicatechin, and isorhamnetin were positively correlated with DPPH. Significant positive associations were observed between chlorogenic acid, rutin and ABTS antioxidant activity, whereas resveratrol displayed an inverse relationship with ABTS. Epicatechin demonstrated a marked positive correlation with CUPRAC, while myricetin showed enhanced affinity toward FRAP. Conversely, kaempferol was negatively correlated with FRAP.

### 3.6. Differences in Volatile Compounds of Wines from Different Regions

**Figure 4:**
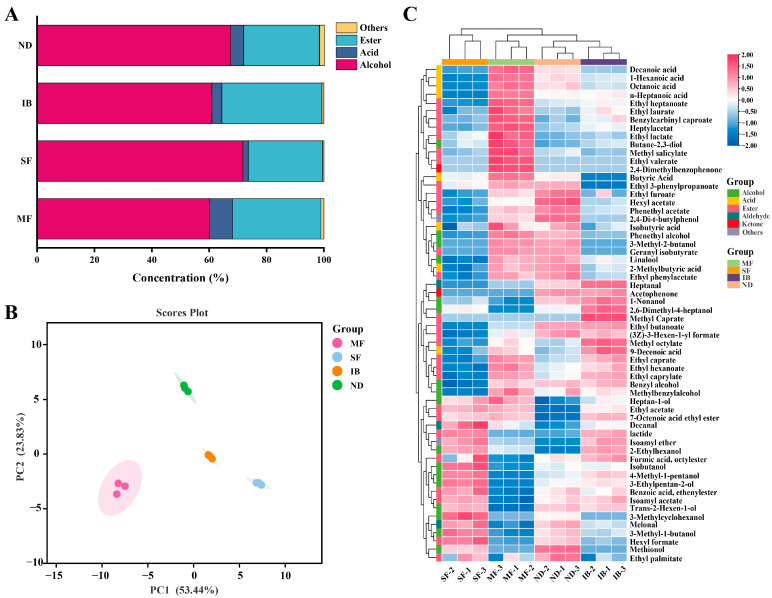
Analysis of all VOCs of wines from different regions. (**A**) Relative composition of VOCs. (**B**) Score plot of principal component analysis (PCA). (**C**) Heat map visualization of all VOCs.

Volatile compounds in wine play an important role in contributing to differences in wine quality [31]. The volatile compounds in wines from four regions were identified by GC-MS/MS, and a total of 60 volatile compounds were identified. The detailed data are shown in Table S3, including alcohols, esters, acids, ketones, aldehydes, ethers, etc. Among them, alcohols and esters accounted for a large proportion of the volatile compounds in the wines of the four regions (Figure 4A), which was similar to the previous research results [13]. Volatile aroma components in wine are mainly represented by several types of alcohols, aldehydes, acids, esters and ketones, and different aromatic compounds can play a role in the characteristic flavor of each type of wine [10].

Alcohols are the main products of alcohol fermentation process, and the main alcohols in the four regional wines are phenylethyl alcohol, 3-methylthiopropanol, isoamyl alcohol and isobutyl alcohol. Phenylethyl alcohol and isoamyl alcohol were significantly different in the four regional wines (*p* < 0.05), which provided floral and fruity aromas for wines [32]. Among the four regions, the content of alcohols in SF region was the highest. Climatic factors will affect the content of glucose and acid, which are the main indica-tors of grape maturity. According to Table S1, the SSC and pH in the SF region were relatively higher in the early stage of fermentation, which may lead to the accumulation of higher alcohols [5].

Ester compounds usually have a positive contribution to the fruity and floral characteristics of wines [33]. A total of 29 esters were detected in wines from four regions. Among them, the contents of isoamyl acetate, ethyl caprylate, phenethyl acetate and ethyl caproate were higher, and there were significant differences in the four regions (*p* < 0.05). It may cause significant differences in flower and fruit aroma in the four regions. This is similar to the results of Chen et al. [34]. The result showed that there were significant differences in floral and fruity aroma characteristics of wines from four sites in Jieshi Mountain, China, mainly related to ethyl esters with higher aroma activity values. The content of ester compounds was significantly higher in the IB region than in the other three regions, which may be affected by temperature and diurnal temperature difference [11]. The greater the diurnal temperature, the higher the activity of lipoxygenase in grape peel, which promoted the synthesis of precursor substances of ester compounds. Therefore, climatic factors can indirectly affect the accumulation of some ester compounds [35].

The volatile acid compounds in wine mainly comes from grape berries [36]. A total of 7 volatile acids were detected in the four regions. The volatile acid content in the MF region was much higher than that in the other three regions. The content of caproic acid and octanoic acid is high, and there are significant differences from the four regional wines (*p* < 0.05), mainly for the wine to bring fat aroma and fruit aroma. The content of volatile acid is also an important index to evaluate wine flavor [37].

Three aldehydes, two ketones, one terpene and ether compounds were also detected in the four regional wines. The content of aldehydes and ketones in MF region is high, and most of the aldehydes and ketones provide floral and fruity aroma for wine. Only linalool was detected in terpenes, and there were significant differences in wines from the four regions (*p* < 0.05), with the highest content in ND region. This may be due to the different intensity of light radiation caused by different altitudes. Some studies have shown that linalool is highly sensitive to sunlight, in which the expression of VviDXS and linalool synthase is reduced by low light intensity, resulting in lower linalool content. It has a floral scent and presents a woody lavender flavor, and terpenes were mostly present in the grape fruit species [38].

In order to understand which volatile compounds varied by region, we performed principal component analysis based on four regions. The differences of volatile components in Cabernet Sauvignon wine affected by intermountain basin system were further analyzed. The two principal components (PC1 and PC2) explained 53.44 % and 23.83 % of the total variability, respectively, and explained 77.27 % of the total variance (Figure 4B). The IB and SF regions are positively distributed on the PC1 axis, and the MF and ND regions are negatively distributed on the PC1 axis, indicating that PC1 may reflect temperature-related indicators. On the PC2 axis, the ND region is positively distributed, and the IB, MF and SF regions are negatively distributed. The PC2 axis may be related to altitude and humidity. The humidity in the four regions gradually decreases with the decrease of altitude. The altitude and humidity in the ND region are the lowest in the four regions. Low altitude and dry environment may affect the accumulation of secondary metabolites through water stress. According to Saito et al., drought stress can activate the ABA signaling pathway in grape peel and promote the activity of phenylalanine ammonia lyase, thereby increasing the synthesis of aromatic compounds such as phenylethanol and benzaldehyde [39]. The results of principal component analysis showed that volatile compounds were significantly different in wines from different regions.

We explored the differences in the accumulation of volatile compounds between wines in different regions by clustering heat map (Figure 4C). Comparative analysis found that most of the volatile compounds in the wines of the four regions were the same, and the volatile compounds could be divided into four clusters according to the similar distribution rules. The first cluster accumulated significantly among MF region. In the second cluster, the accumulation of MF and ND regions was higher. The third cluster was significantly accumulated in IB region, and the fourth cluster was significantly accumulated among SF region.

### 3.7. Analysis of Characteristic Volatile Compounds

The difference in volatile compounds between different wines is largely due to the different content of volatile compounds [11]. Although there are many kinds of volatile compounds in wine, in fact, only a few characteristic volatile compounds are helpful to perceive the aroma of wine and contribute to the overall aroma of wine [40]. In this study, there were 11 volatile compounds with OAV > 1 (Table 2), which were the main characteristic volatile compounds of wines in four regions.

**Figure 5:**
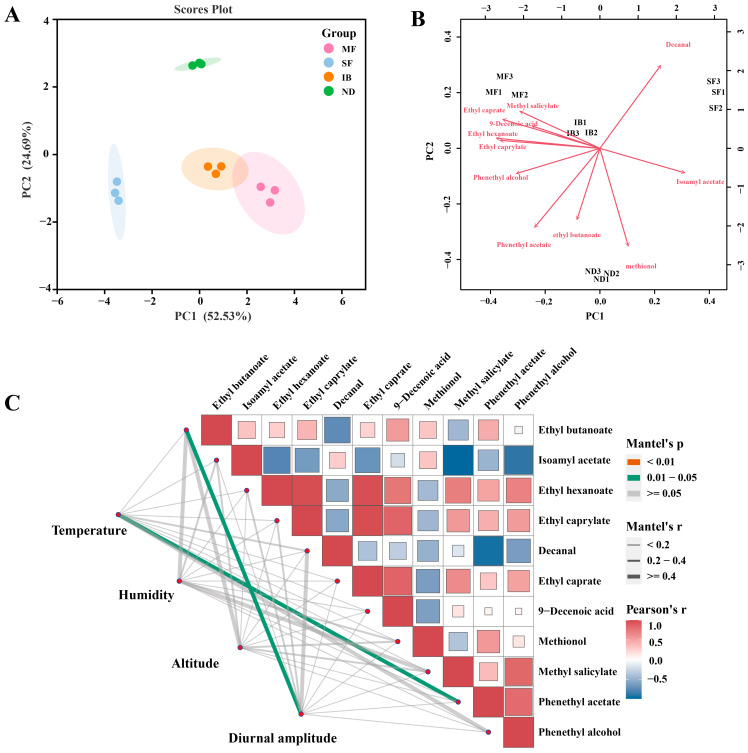
Multivariate statistical analysis of characteristic volatile compounds. (**A**) Principal component analysis of characteristic volatile compounds (VIP > 1). (**B**) Loading plot of Characteristic volatile compounds (VIP > 1). (**C**) The correlation between meteorological factors and characteristic aroma compounds was analyzed by cluster heat map and network map.

The effects of mountain-basin system on these volatile compounds in wines from different regions were studied based on principal component analysis (Figure 5A). The two principal components (PC1 and PC2) explained 52.53 % and 24.69 % of the total variability, respectively. It can be seen from the diagram that different samples have a high degree of separation, and there are significant differences in volatile components in the four regions. From the load diagram (Figure 5B), the IB and MF regions are in the same quadrant, and the SF and ND regions are in the other two quadrants. Methyl salicylate, ethyl caprate and ethyl hexanoate had strong correlation with MF and IB regions, which contributed to the characteristics of holly leaf aroma and fruit aroma for wine. Decanal is the main characteristic aroma in the SF region, which gives the wine citrus peel odor. Ethyl butyrate and 3-methylthiopropanol were the main characteristic aromas in the ND region, which had the odors of pineapple, banana and apple. The correlation between meteorological factors and characteristic aroma compounds was analyzed by the combination of cluster heat map and network map (Figure 5C). The analysis showed that the ambient temperature was significantly correlated with the concentration of phenethyl acetate, while the diurnal temperature change was proportional to the concentration of ethyl butyrate in the vineyard. The altitude and humidity had a strong correlation with ethyl butyrate, the humidity had a strong correlation with methyl salicylate and phenethyl alcohol, and the diurnal amplitude had a strong correlation with decanal.

## 4. Conclusions

This study focused on the impact of mountain-basin system characteristics on wine quality. By comparing physicochemical properties, phenolics, antioxidant activity and volatile compounds of wines from distinct regions, it elucidated the regulatory mechanisms of climatic gradient variations on wine quality. With the change of altitude, the temperature and humidity of the four regions are significantly different. There were significant differences in the physicochemical index, antioxidant capacity and volatile compound content of wines in the four regions affected by the climate environment. Analyses revealed gradient variations in alcohol content, soluble solids, and pH values across regions, while residual sugar and titratable acidity showed relatively minor differences. Among organic acids, malic acid content demonstrated particular sensitivity to altitude and temperature variations. Phenolic analysis indicated significant regional disparities in total phenols, flavonoids, tannins, and anthocyanins. The monomeric phenols in the four regions were significantly correlated with antioxidant activity, and the IB region showed strong antioxidant capacity. In addition, 60 volatile compounds were identified in wines from the four regions, of which 11 were key volatile compounds in the wines. Key volatile compounds included methyl salicylate, ethyl decanoate, and ethyl hexanoate in MF and IB regions. Decanal as the signature aroma in SF region, while ND region exhibited a unique flavor profile dominated by ethyl butyrate and 3-methylthiopropanol. In this study, the meteorological indexes were combined with the basic physicochemical and characteristic aroma of wine to find the correlation between climate and wine quality and flavor. It proves the significance of fine classification of small wine producing areas, and provides a theoretical basis for the subsequent regionalization construction of grape and wine industry in Xinjiang.

## Figures and Tables

**Table 1:** Differences of meteorological indicators in different regions (mg·L^−1^).

Characteristics	MF	SF	IB	ND
Temperature (°C)	18.45 ± 0.20 b	17.27 ± 0.11 d	17.57 ± 0.26 c	18.81 ± 0.01 a
Humidity (%)	68.99 ± 2.31 a	67.79 ± 1.43 b	67.21 ± 0.82 c	66.69 ± 0.89 d
Diurnal amplitude (°C)	19.32 ± 0.16 c	18.86 ± 0.12 d	19.88 ± 0.23 a	19.67 ± 0.1 b
Altitude (m)	617	492	384	296

Notes: The data were expressed as mean ± standard deviation of three replicates. The letters indicate significant between different regions (*p* < 0.05).

**Table 2:** OAV value and flavor description of main aroma compounds in wines from different regions.

Volatile Compounds	RI	Threshold (µg/L)	MF	SF	IB	ND	Flavor Description
Ethyl butanoate	1021	20	3.48	0	27.06	24.22	Apple, pineapple
Isoamyl acetate	1115	30	188.09	278.63	268.63	256.29	Banana, pear
Ethyl hexanoate	1238	5	482.69	281.11	431.33	374.73	Pineapple, banana
Ethyl caprylate	1440	580	891.07	565.2	872.37	731.68	Brandy
Decanal	1496	0.1	6.2	8.14	6.15	4.79	Citrus peel
Ethyl caprate	1625	122	161.01	78.27	149.9	104.16	Pear, brandy
Methionol	1715	8	0	3.62	0	11.33	Onion, meat stink
Methyl salicylate	1730	40	43.54	19.24	19.21	21.92	Medicinal herb
Phenethyl acetate	1804	98	105.64	64.87	84.93	125.73	Rose, honey
Phenethyl alcohol	1920	1000	1881.52	667.07	820.54	1508.46	Rose
9-Decenoic acid	2305	4.3	87.77	70.06	101.7	76.84	Fatty, wax fragrance

Notes: The data were expressed as mean deviation of three replicates (*n* = 3). Odor thresholds with reference to relevant literature [13,41,42].

## Data Availability

The original contributions presented in this study are included in the article/Supplementary Material. Further inquiries can be directed to the corresponding authors.

## Supplementary Materials

The following supporting information can be downloaded at: https://www.mdpi.com/article/10.3390/foods14071086/s1, Table S1: Changes of soluble solids and pH during wine fermentation; Table S2: Contents of phenolic compounds in wines from different regions (mg/L); Table S3 Contents of volatile components in wines from different regions (µg/L).
