# Supplementary material for: Effects of Mountain-Basin System on Chemical Composition, Antioxidant Activity and Volatile Flavor Substances of Cabernet Sauvignon Wines in Xinjiang Region, China
Source: Foods. 2025 Mar 21;14(7):1086. doi: 10.3390/foods14071086 (PMC11988972; doi:10.3390/foods14071086)
Supplement: Supplementary file 1 [file foods-14-01086-s001.zip › foods-3498668-supplementary.pdf]

Table S1 Changes of soluble solids and pH during wine fermentation

	MF		SF		IB		ND	
Days	SSC	pH	SSC	pH	SSC	pH	SSC	pH
0d	22.6	3.77	23.7	4.08	23.4	4.01	22.1	3.84
1d	20.9	3.68	22.4	3.97	21.3	3.90	20.1	3.80
2d	15.2	3.60	16.7	3.80	15	3.75	14.4	3.64
3d	9.9	3.64	11.3	3.90	9.4	3.86	9.2	3.77
4d	6.9	3.67	8	3.94	7	3.92	6.6	3.79
5d	6.3	3.74	7.1	4	6.9	3.99	6.5	3.85
6d	6.2	3.73	7.1	3.99	6.8	3.98	6.5	3.83
7d	6.2	3.71	7.1	3.99	6.8	3.97	6.5	3.83

Notes: 0d is the initial soluble solids content and pH of the grape juice, and the soluble solids content and 7d is the soluble solids content and pH of the wine after the fermentation.

Table S2 Contents of phenolic compounds in wines from different regions (mg/L)

	MF	SF	IB	ND
Trans-Ferulic acid	22.24±0.56^{c}	23.63±1.07^{b}	32.71±1.60^{a}	21.07±0.84^{d}
Gallic acid	12.81±0.32^{c}	13.06±0.59^{c}	18.45±0.90^{a}	16.27±0.65^{b}
Neochlorogenic acid	0.53±0.01^{a}	0.09±0.01^{d}	0.17±0.01^{b}	0.12±0.01^{c}
Catechin	48.41±1.22^{a}	21.66±0.98^{c}	38.09±1.86^{b}	16.94±0.68^{d}
Vanillic acid	4.63±0.12^{a}	2.29±0.10^{d}	3.31±0.16^{b}	2.84±0.11^{c}
Syringic acid	nd	nd	1.84±0.09^{a}	1.19±0.05^{b}
Chlorogenic acid	2.75±0.07^{c}	6.98±0.31^{b}	9.49±0.46^{a}	nd
Epicatechin	38.92±0.98^{c}	34.98±1.58^{d}	58.22±2.85^{b}	78.33±3.13^{a}
Caffeic acid	9.22±0.23^{b}	2.91±0.13^{d}	9.50±0.46^{a}	7.46±0.30^{c}
Ferulic acid	1.75±0.04^{d}	8.14±0.37^{a}	7.44±0.36^{b}	3.04±0.12^{c}
Benzoic acid	5.18±0.13^{d}	9.08±0.41^{a}	8.47±0.41^{b}	7.36±0.30^{c}
Rutin	45.00±1.13^{d}	60.85±2.74^{b}	72.36±3.54^{a}	58.05±2.32^{c}
Resveratrol	2.09±0.05^{b}	nd	1.58±0.08^{c}	2.46±0.10^{a}
Myricetin	nd	6.70±0.30^{c}	8.89±0.43^{a}	7.92±0.32^{b}
Quercetin	nd	nd	0.69±0.03^{a}	0.62±0.02^{b}
Isorhamnetin	8.37±0.21^{b}	8.26±0.37^{b}	9.39±0.46^{a}	8.18±0.33^{b}
Kaempferol	2.65±0.07^{b}	3.81±0.17^{a}	1.76±0.09^{d}	2.11±0.08^{c}

Notes: Data are expressed as the mean±standard deviation from replicate analyses (n = 3) of three replicate samples. The different lowercase letters in each row indicate significant differences between samples from different regions ($p < 0.05$). The "nd" indicates that it is not detected.

Table S3 Contents of volatile components in wines from different regions (μg/L)

	Compounds	RI	MF	SF	IB	ND
	**Alcohol**					
A1	3-Methyl-2-butanol	1094	$0.85±0.03^{a}$	nd	nd	$0.80±0.05^{b}$
A2	Isobutanol	1097	$159.17±4.35^{c}$	$399.05±28.05^{a}$	$237.2±6.03^{b}$	$236.12±11.22^{b}$
A3	isoamyl alcohol	1221	$2548.23±72.16^{d}$	$3941.47±101.14^{a}$	$2983.65±73.21^{c}$	$3449.84±110.78^{b}$
A4	4-Methyl-1-pentanol	1299	$0.84±0.01^{d}$	$3.62±0.2^{a}$	$2.58±0.15^{b}$	$1.81±0.11^{c}$
A5	2-Pentanol, 3-ethyl-	1330	nd	$1.69±0.1^{a}$	$0.85±0.04^{b}$	$0.64±0.05^{c}$
A6	Trans-2-Hexen-1-ol	1390	nd	$1.36±0.05^{a}$	$1.17±0.08^{b}$	$1.24±0.08^{b}$
A7	Heptan-1-ol	1460	$103.29±6.8^{a}$	$96.56±5.08^{b}$	$87.64±3.57^{c}$	$71.18±3.66^{d}$
A8	2-Ethylhexanol	1492	$2.48±0.16^{b}$	$5.65±0.35^{a}$	$5.62±0.32^{a}$	$0.74±0.02^{c}$
A9	3-Methylcyclohexanol	1502	nd	$0.79±0.08^{a}$	nd	$0.26±0.01^{b}$
A10	2,6-Dimethyl-4-heptanol	1509	nd	$0.96±0.13^{b}$	$2.8±0.27^{a}$	$0.86±0.02^{b}$
A11	Butane-2,3-diol	1583	$8.65±0.62^{a}$	$4.74±0.37^{b}$	$4.78±0.2^{b}$	$3.91±0.15^{c}$
A12	1-Nonanol	1640	$5.37±0.28^{d}$	$6.77±0.44^{c}$	$10.98±0.56^{a}$	$9.22±0.42^{b}$
A13	Methionol	1715	nd	$3.62±0.52^{b}$	nd	$11.33±0.66^{a}$
A14	Benzyl alcohol	1880	$5.97±0.31^{a}$	$2.54±0.17^{b}$	$6.38±0.44^{a}$	$6.25±0.26^{a}$
A15	Phenethyl alcohol	1920	$1881.52±97.28^{a}$	$667.07±13.66^{d}$	$820.54±18.64^{c}$	$1508.46±29.54^{b}$
A16	Methylbenzylalcohol	1955	$0.94±0.1^{a}$	$0.32±0.01^{c}$	$0.72±0.04^{b}$	$0.73±0.16^{b}$
	Total		4717.31	5136.21	4164.91	5303.39
	**Acids**					
B1	Isobutyric acid	1581	$7.95±0.53^{a}$	$6.69±0.47^{b}$	$6.95±0.3^{b}$	$7.74±0.32^{a}$
B2	Butyric Acid	1613	$2.17±0.08^{a}$	$0.8±0.11^{c}$	nd	$0.97±0.04^{b}$
B3	2-Methylbutyric acid	1657	$29.3±1.32^{b}$	$17.03±1.33^{d}$	$22.13±1.36^{c}$	$31.27±1.13^{a}$
B4	Caproic acid	1841	$153.63±3.98^{a}$	$34.91±1.6^{d}$	$64.87±2.57^{c}$	$88.9±4.66^{b}$
B5	n-Heptanoic acid	1915	$2.5±0.06^{a}$	nd	$1.05±0.11^{b}$	$1.03±0.04^{b}$
B6	Octanoic acid	2022	$370.64±24.89^{a}$	$63.23±2.47^{d}$	$125.99±5.97^{c}$	$203.13±18.94^{b}$
B7	Decanoic acid	2281	$52.87±3.12^{a}$	$10.24±0.32^{d}$	$14.24±0.35^{c}$	$25.97±0.53^{b}$
	Total		619.06	132.9	235.23	359.01

	**Ester**					
C1	Ethyl acetate	899	215.72±7.78^{a}	223.92±15.74^{a}	180.28±3.99^{b}	89.96±1.29^{c}
C2	Ethyl butanoate	1021	3.48±0.12^{c}	nd	27.06±1.96^{a}	24.22±0.56^{b}
C3	Isoamyl acetate	1115	188.09±5.4^{c}	278.63±2.96^{a}	268.63±11.68^{a}	256.29±8.4^{b}
C4	Lactide	1198	4.97±0.14^{c}	15.29±0.37^{a}	12.9±0.56^{b}	4.61±0.19^{c}
C5	Ethyl hexanoate	1238	482.69±19.92^{a}	281.11±8.95^{d}	431.33±25.54^{b}	374.73±12.69^{c}
C6	Benzylcarbinyl caproate	1251	1.87±0.05^{a}	0.77±0.04^{c}	1.12±0.14^{b}	0.78±0.08^{c}
C7	Hexyl acetate	1269	13.95±0.38^{b}	7.71±0.87^{d}	11±0.75^{c}	29.7±1.71^{a}
C8	Vinyl benzoate	1275	86.43±2.4^{c}	157.76±13.53^{a}	126.12±9.06^{b}	133.42±7.74^{b}
C9	Hexyl formate	1302	3.6±0.1^{c}	10.2±0.91^{a}	4.34±0.23^{c}	7.18±0.36^{b}
C10	Ethyl heptanoate	1327	25.72±0.85^{a}	6.98±0.16^{d}	12.85±0.8^{b}	11.4±0.53^{c}
C11	Heptylacetat	1370	8.78±0.25^{a}	3.49±0.22^{d}	4.83±0.13^{b}	4.06±0.21^{c}
C12	(3Z)-3-Hexen-1-yl formate	1380	1.27±0.08^{c}	0.83±0.03^{d}	1.48±0.04^{b}	1.59±0.08^{a}
C13	Methyl octylate	1387	2.21±0.12^{b}	1.5±0.05^{d}	3.00±0.08^{a}	1.89±0.10^{c}
C14	Ethyl caprylate	1440	891.07±14.84^{a}	565.2±15.74^{d}	872.37±14.55^{b}	731.68±23.98^{c}
C15	Ethyl valerate	1138	0.15±0.01^{a}	nd	nd	nd
C16	7-Octenoic acid ethyl ester	1486	8.76±0.58^{a}	8.19±0.44^{b}	9.09±0.31^{a}	2.78±0.11^{c}
C17	Ethyl lactate	1505	40.05±3.63^{a}	23.11±1.71^{b}	21.19±0.73^{b}	19.4±0.8^{b}
C18	Formic acid, octylester	1560	12.77±0.51^{b}	19.2±5.47^{a}	20.91±0.89^{a}	18.00±0.57^{a}
C19	Methyl Caprate	1590	nd	nd	2.51±0.05^{a}	nd
C20	Ethyl furoate	1606	0.23±0.01^{b}	0.13±0.01^{d}	0.18±0.05^{c}	0.29±0.01^{a}
C21	Ethyl caprate	1625	161.01±5.19^{a}	78.27±6.10^{d}	149.9±10.33^{b}	104.16±3.93^{c}
C22	Methyl salicylate	1730	43.54±2.95^{a}	19.24±1.17^{c}	19.21±0.70^{c}	21.92±1.29^{b}
C23	Ethyl phenylacetate	1765	5.12±0.35^{b}	2.76±0.17^{d}	3.6±0.15^{c}	5.52±0.33^{a}
C24	Geranyl isobutyrate	1790	1.88±0.13^{a}	nd	nd	1.7±0.03^{b}
C25	Phenethyl acetate	1804	105.64±3.13^{b}	64.87±2.85^{d}	84.93±2.26^{c}	125.73±4.06^{a}
C26	Ethyl laurate	1825	23.55±1.46^{a}	14.00±1.13^{c}	17.74±0.89^{c}	15.35±0.27^{c}
C27	Ethyl 3-phenylpropanoate	1908	1.54±0.08^{a}	0.85±0.13^{b}	nd	1.48±0.01^{a}
C28	Ethyl palmitate	2250	0.96±0.06^{bc}	1.02±0.07^{ab}	0.93±0.04^{c}	1.08±0.03^{a}
C29	9-Decenoic acid	2305	87.77±3.57^{b}	70.06±4.28^{d}	101.7±4.23^{a}	76.84±2.76^{c}

	Total		2422.82	1855.09	2389.2	2065.76
	**Aldehydes and Ketones**					
D1	Heptanal	1206	0.2±0.01^{c}	nd	1.1±0.02^{a}	0.62±0.02^{b}
D2	Melonal	1330	3.37±0.2^{c}	5.96±0.44^{a}	4.37±0.1^{b}	5.49±0.28^{a}
D3	Decanal	1496	6.2±0.4^{b}	8.14±0.84^{a}	6.15±0.18^{b}	4.79±0.17^{c}
D4	2,4-Dimethylbenzophenone	1511	8.78±0.56^{a}	nd	nd	nd
D5	Acetophenone	1628	nd	nd	0.73±0.08^{a}	0.73±0.03^{a}
	Total		18.55	14.1	12.35	11.63
	**Others**					
E1	Linalool	1547	2.61±0.15^{a}	1.12±0.07^{c}	1.95±0.15^{b}	2.68±0.11^{a}
E2	Isoamyl ether	1067	0.11±0.01^{b}	0.37±0.05^{a}	0.33±0.05^{a}	nd
E3	2,4-Di-t-butylphenol	2316	62.57±2.88^{b}	22.07±1.31^{d}	33.85±0.26^{c}	118.43±2.42^{a}
	Total		65.29	23.56	36.13	121.11

Notes: The data were expressed as mean ± standard deviation of repeated analysis of three replicate samples (n = 3). Different lowercase letters in each line represent significant differences between samples ($p < 0.05$). The "nd" indicates that it is not detected.
